# Feasibility and Acceptability of a Brief Psychological Sleep Intervention (APS) for Depressed Psychiatric Inpatients: A Pilot Study

**DOI:** 10.1192/j.eurpsy.2026.11985

**Published:** 2026-07-31

**Authors:** S. Tamm, J. Stenfalk, M. Troili, K. Blom, S. Jernelöv

**Affiliations:** 1 Karolinska Institutet, Stockholm; 2 Region Uppsala, Uppsala, Sweden

## Abstract

**Introduction:**

Sleep problems are highly prevalent among psychiatric inpatients and represent a negative prognostic factor for psychiatric symptoms, affective episodes, and suicidal behaviors. While Cognitive Behavioral Therapy for Insomnia (CBT-I) is the first-line outpatient treatment, it is rarely offered in inpatient settings due to organizational factors and short stays. Research on effective inpatient sleep management is limited. This study evaluated a novel, brief psychological intervention, Acute Psychological Sleep Stabilisation (APS), designed for this context.

**Objectives:**

To evaluate the feasibility (primary outcome) and acceptability of APS among psychiatric inpatients with depression and sleep problems, and to calculate preliminary treatment effects to inform future power calculations for a randomized controlled trial (RCT).

**Methods:**

In this prospective pilot study, 20 patients admitted to psychiatric inpatient wards with diagnosed depression and comorbid sleep problems received APS, a 4-session intervention. Feasibility was defined a priori as attrition < 50% before day 14 post-enrollment. Acceptability was assessed using the Treament Credibility Scale. Secondary outcomes included changes in insomnia severity (Insomnia Severity Index, ISI) and depressive symptoms (Patient Health Questionnaire-9, PHQ-9) from pre- to post-intervention (14 days post-enrollment), sleep parameters (via sleep diary and actigraphy), and length of stay.

**Results:**

Feasibility criteria were met, with attrition at 10% (2/20 participants) before day 14. Acceptability ratings were high. Significant improvements were observed from pre- to post-intervention: mean ISI decreased from 20.1 to 13.6 (within-group Cohen’s d = 1.16), and mean PHQ-9 decreased from 19.4 to 12.1. Median sleep efficiency derived from sleep diaries at day 7 was 87.7%. Mean time until discharge was 22.7 days.

**Conclusions:**

APS demonstrates feasibility and high acceptability as a brief intervention for sleep problems in acutely ill, depressed psychiatric inpatients. Preliminary data show clinically relevant reductions in both insomnia and depressive symptoms, with effect sizes comparable to previous studies of more intensive interventions. These promising findings strongly support proceeding to a larger RCT to definitively evaluate the efficacy of APS in this vulnerable population.

**Disclosure of Interest:**

None Declared

